# Topology and porosity control of metal–organic frameworks through linker functionalization†
†Electronic supplementary information (ESI) available: Proton NMR spectra of organic ligands and MOF materials, crystallographic data, TGA curves, DRIFTS spectra and SEM images are included in the ESI. CCDC [1854453 and 1855836]. For ESI and crystallographic data in CIF or other electronic format see DOI: 10.1039/c8sc04220a


**DOI:** 10.1039/c8sc04220a

**Published:** 2018-11-09

**Authors:** Jiafei Lyu, Xuan Zhang, Ken-ichi Otake, Xingjie Wang, Peng Li, Zhanyong Li, Zhijie Chen, Yuanyuan Zhang, Megan C. Wasson, Ying Yang, Peng Bai, Xianghai Guo, Timur Islamoglu, Omar K. Farha

**Affiliations:** a Department of Pharmaceutical Engineering , School of Chemical Engineering and Technology , Tianjin University , Tianjin 300350 , China; b Key Laboratory of Systems Bioengineering , Ministry of Education , Tianjin University , Tianjin 300350 , China; c Department of Chemistry and International Institute of Nanotechnology , Northwestern University , 2145 Sheridan Road , Evanston , Illinois 60208 , USA . Email: o-farha@northwestern.edu

## Abstract

Tetratopic organic linkers have been extensively used in Zr-based metal–organic frameworks (MOFs) where diverse topologies have been observed. Achieving meticulous control over the topologies to tune the pore sizes and shapes of the resulting materials, however, remains a great challenge. Herein, by introducing substituents to the backbone of tetratopic linkers to affect the linker conformation, phase-pure Zr-MOFs with different topologies and porosity were successfully obtained under the same synthetic conditions. The conversion of CO_2_ to valuable cyclic carbonates is a promising route for the mitigation of the greenhouse gas. Owing to the presence of substrate accessible Lewis acidic Zr(iv) sites in the 8-connected Zr_6_ nodes, the Zr-MOFs in this study have been investigated as heterogenous acid catalysts for CO_2_ cycloaddition to styrene oxide. The MOFs exhibited drastically different catalytic activities depending on their distinct pore structures. Compared to previously reported MOF materials, a superior catalytic activity was observed with the mesoporous NU-1008, giving an almost 100% conversion under mild conditions.

## Abstract 2

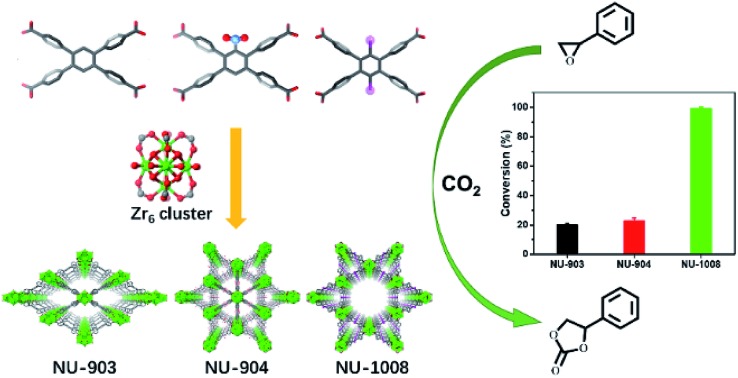
Topology and porosity control of Zr6-based MOFs was achieved by introducing steric functionalization into the conformations of substituted tetracarboxylate linkers.

## Introduction

Metal–organic frameworks (MOFs),^1^–^3^ a class of crystalline porous materials constructed from metal nodes and organic ligands by coordination bonds, have found a wide range of applications such as gas storage and separation,^4^–^11^ catalysis,^12^–^16^ magnetism,^17^,^18^ drug delivery,^19^–^23^ small-molecule recognition^24^ and chemical sensing.^25^,^26^ This functional diversity is largely due to their versatile structural tunability where a variety of components can be combined to generate an almost indefinite number of novel materials. However, elucidating the parameters that dictate the MOF structures, including their topology and porosity, is non-trivial. Considering Zr_6_ cluster-based MOFs as an example, although linkers of lower connectivity generally favor one particular topology,^27^–^29^ more topologies can be accessed from linkers with higher connectivity^30^,^31^ due to their tendency to undergo conformational changes, resulting in challenges in predicting MOF structures.^32^–^34^

Since the first reports of Zr-MOFs with tetratopic linkers,^35^–^37^ several topologies have been observed, including **ftw**,^36^,^38^–^41^ **csq**,^35^–^37^,^42^ **she**,^43^**shp**,^44^**scu**,^32^,^45^ **flu**,^27^,^46^,^47^ **ith**,^27^**sqc**,^48^**lvt**^49^ and **stp**^50^ which can be attributed to the linker conformation adjustments under different synthetic conditions. This conformational change can be induced by controlling one of the many factors, such as the modulating reagent, concentration, metal salt, temperature and solvent. For example, a **csq**-net MOF (NU-1000)^42^,^51^ is produced when using benzoic acid as the modulator whereas a **scu**-net MOF (NU-901)^32^ is obtained using 4-amino-benzoic acid with the same 1,3,6,8-tetrakis(*p*-benzoic acid)pyrene (TBAPy) linker and Zr precursor. Using a tetratopic linker with arms of a high degree of rotational freedom, such as tetracarboxyphenylporphyrin (TCPP), multiple topologies have been observed.^35^–^37^,^43^,^44^,^48^ A similar phenomenon was also observed when we explored the isoreticular tetracarboxylate Zr-MOF **csq**-net NU-100X series for enzyme immobilization where MOFs with **ftw** topology were produced instead when using linkers with longer arms. Therefore, our group concluded that the torsion angle between the planar “backbone” (benzene, pyrene, porphyrin) and the arms (carboxylic acid) had a critical influence on the framework topology. Specifically, if the torsion angle was close to 60°, the **csq** topology was favored, whereas the **ftw** topology formed if the torsion angle was close to 0°.^52^

In addition to using organic linkers with higher rotational freedom, installing functional groups to the organic linkers affords another effective strategy to affect the linker conformation, thereby the material topology. For instance, Yaghi and co-workers successfully synthesized **qom**-net MOF-177 series with uncommon topologies (**pyr** and **rtl**) by introducing functional groups.^53^ Recently, Zhou and co-workers introduced steric hinderance into the biphenyl-3,3′,5,5′-tetra(phenyl-4-carboxylic acid) (TPCB) linker to affect linker conformation, which subsequently effect the topology of the resulting Zr-tetracarboxylate MOFs. However, accompanying the original structure were mixed phase materials that formed as byproducts, most likely due to the introduction of the bulky substituents.^54^ In high agreement with experimental results, computational studies compared the energy of MOFs with different substituents to demonstrate the influence of introduced steric hinderance on linker conformation and MOF topology.^55^ With these design rules and challenges in mind, we set out to tune the steric hindrance of the organic linkers in a stepwise fashion, aiming to achieve topology control in the resulting series of Zr-MOFs.

## Results and discussion

### Introduction of steric hinderance

**Fig. 1:**
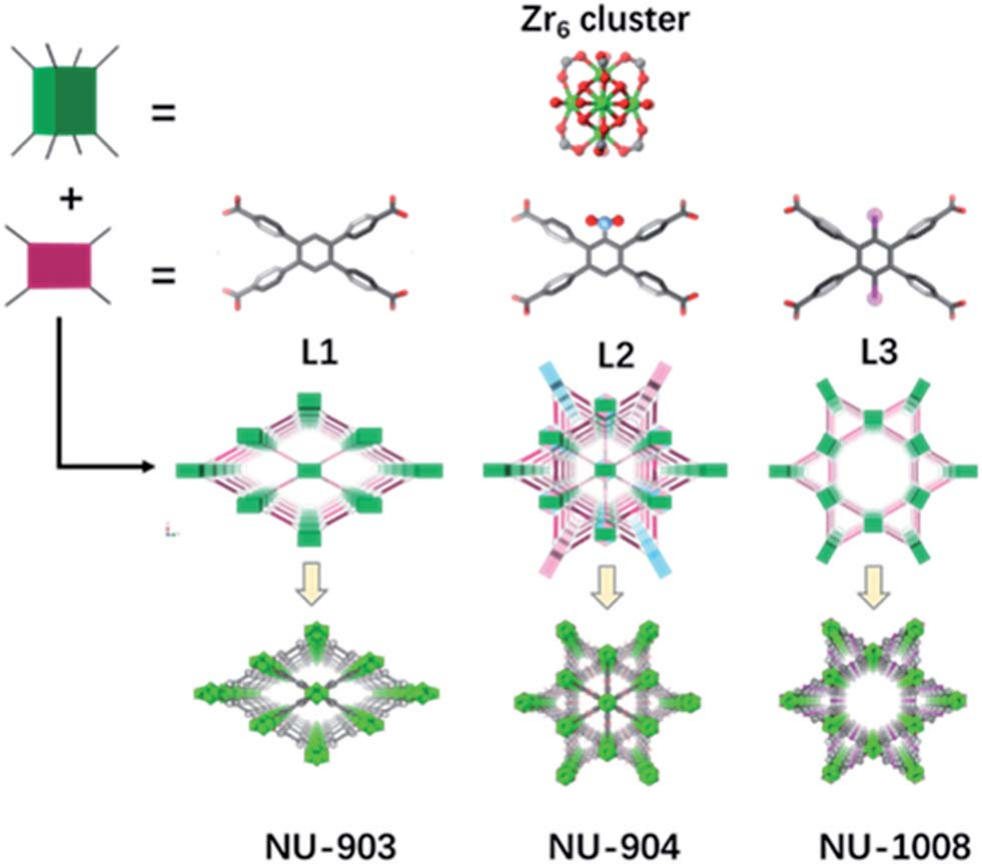
Schematic representation of the construction of NU-903, NU-904 and NU-1008. Atom colour scheme: C, grey; N, blue; O, red; Br, pink; Zr, green polyhedra. H atoms are omitted for clarity.

In addition to the 1,2,4,5-tetrakis(4-carboxyphenyl)benzene (TCPB) linker (Fig. 1, L1), two more linkers were designed. A nitro group (Fig. 1, L2) and two bromo groups (Fig. 1, L3) were installed on the central benzene respectively to introduce steric hindrance to affect the rotational freedom of the peripheral benzoate arms and in turn to control the topology of the resulting MOFs. To ensure consistency and to eliminate variability from factors such as solvent, temperature and modulator on the topology of the resulting MOFs, the syntheses with the three TCPB-based linkers were carried out under identical solvothermal conditions (ESI†).

### Topology and porosity diversity of the as-synthesized MOFs

As previously reported by Stock and co-workers,^45^ Zr_6_ nodes and the L1 linker form NU-903 with an **scu** topology with characteristic diamond-shaped channels along the *c* axis (Fig. 1). NU-903 is isoreticular to TBAPy-based NU-901 32 and TCPP-based NU-902.^56^

**Fig. 2:**
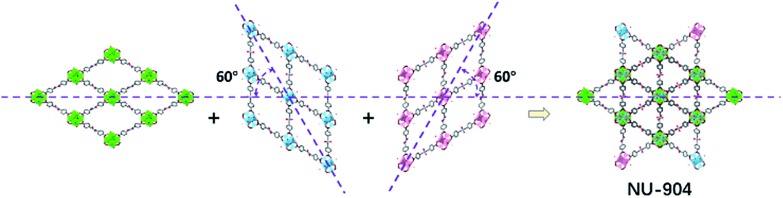
The reticular twin structure of NU-904.

**Fig. 3:**
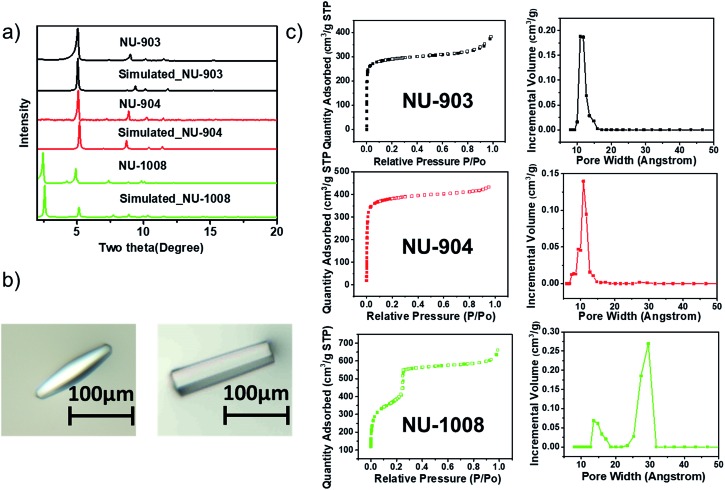
Characterization of the three MOFs. (a) PXRD patterns; (b) optical images of the single crystals of NU-904 (left) and NU-1008 (right), the scale bars in the images are 100 μm; (c) N_2_ sorption isotherms and DFT pore size distribution of NU-903, NU-904 and NU-1008.

The crystals of the resulting MOF with linker L2 are oval in shape (Fig. 3b). Single crystal X-ray diffraction analysis (Table S1, ESI†) revealed that NU-904 crystallized in the *P*2/*m* space group (*a* = 19.64 (1) Å, *b* = 12.63 (1) Å, *c* = 19.635 (4) Å and *β* = 119.994° (2) at 200 K) with the formula as Zr_6_(μ-O)_4_(μ-OH)_4_(HCOO)_1.5_(H_2_O)_2.5_(OH)_2.5_(L2)_2_. The 3D structure consists of 8-connected Zr_6_ nodes and mononitro-substituted TCPB linkers, yielding a rare **scu** topology.^32^ The single crystal structure of NU-904 is characteristic of a reticular-merohedral twin structure in which three orientations stack together along the *b* axis, twisting 60° from each other (Fig. 2). The reticular twins of three **scu**-net components gave rise to the overall 6-fold symmetry in the structure. Topologically, the average structure of the threefold twinned NU-904 is based on the highly connected 4,12-c **shp** net.^44^,^57^ Interestingly, the threefold twins of ordered 4,8-c **scu** structure led to a twinned 4,12-c rare **shp** structure (Fig. S17, ESI†). To the best of our knowledge, the inherent correlation between these two topological nets has not been realized until this work. As a result, the average structure is observed as triangular-shaped instead of the expected diamond-shaped channels in NU-903 (Fig. 2).

The MOF synthesis with linker L3 yields hexagonal rod-shaped crystals (Fig. 3b). Single crystal X-ray diffraction analysis (Table S1, ESI†) revealed that NU-1008 crystallized in the hexagonal *P*6/*mmm* space group with the chemical formula as Zr_6_(μ-O)_4_(μ-OH)_4_(HCOO)(H_2_O)_3_(OH)_3_(L3)_2_; the 3D structure consists of 8-connected Zr_6_ nodes and dibromo-substituted TCPB linkers in a **csq** topology. Isoreticular to NU-1000,^58^ there are two types of channels along the *c* axis, a 1 nm wide triangular channel and a 3 nm hexagonal channel (Fig. 1). The incorporation of the dibromo groups does not compromise the porosity of the material because the bromo groups reside in the window connecting the hierarchically triangular micropores and hexagonal mesopores. Therefore, the pore size distribution of NU-1008 is similar to NU-1000.

### Characterization of as-synthesized MOFs

Due to the 8-connected Zr_6_ nodes present in all three MOFs, ^1^H NMR was employed to identify the coordinated species on the remaining accessible sites of the node. Upon digestion of the MOFs with a dilute NaOD solution, a peak was observed for all three materials around 8.37 ppm that is characteristic of formate ligand. Quantifying the amounts of formate relative to the organic linker in the three samples gives *ca.* 2, 2 and 3 formate groups per Zr_6_ node in NU-903 (Fig. S4, ESI†), NU-904 (Fig. S5, ESI†) and NU-1008 (Fig. S6, ESI†), respectively. Diffuse reflectance infrared fourier transform spectroscopy (DRIFTS) also confirms the presence of formate groups with the observed C–H stretching at 2745 cm^–1^. In addition, N=O stretching (1658 and 1373 cm^–1^) and C–Br stretching (712 cm^–1^) in the DRIFTS spectra of NU-904 and NU-1008 confirmed the presence of L2 and L3, respectively (Fig. S10–S12, ESI†).

The phase purity of the bulk materials was confirmed by PXRD (Fig. 3a). The particle morphology of NU-904 is oval-shaped and NU-1008 is hexagonal rod-shaped (Fig. 3b), as evidenced by the SEM images (Fig. S13, ESI†). Nitrogen sorption isotherms of three different MOFs were measured at 77 K; the type I isotherms of NU-903 and NU-904 are indicative of microporous structure of the MOFs, while the type IV isotherm of NU-1008 indicates the presence of both micropores and mesopores in the material. The BET areas were calculated to be 1140, 1410 and 1400 m^2^ g^–1^ for NU-903, NU-904 and NU-1008, respectively. DFT pore size distributions reveal micropores of 11 Å for NU-903, 10 Å for NU-904 and hierarchical micropores of 11 Å and mesopores of 29 Å for NU-1008 (Fig. 3c). The total pore volumes were 0.515, 0.613 and 0.819 cm^3^ g^–1^ for NU-903, NU-904 and NU-1008, respectively. The larger gravimetric pore volume and the type IV isotherm of NU-1008 is again consistent with a mesoporous structure. Thermogravimetric analysis (TGA) under air showed no sign of mass loss up to 400 °C (Fig. S7–S9, ESI†), demonstrating the high thermal stability of the three MOFs. In addition, all three materials showed good stability under acidic condition with treatment of 0.5 M HCl aqueous solution for 10 hours (Fig. S14, ESI†).

### Topology and porosity control through introduction of steric effect

**Fig. 4:**
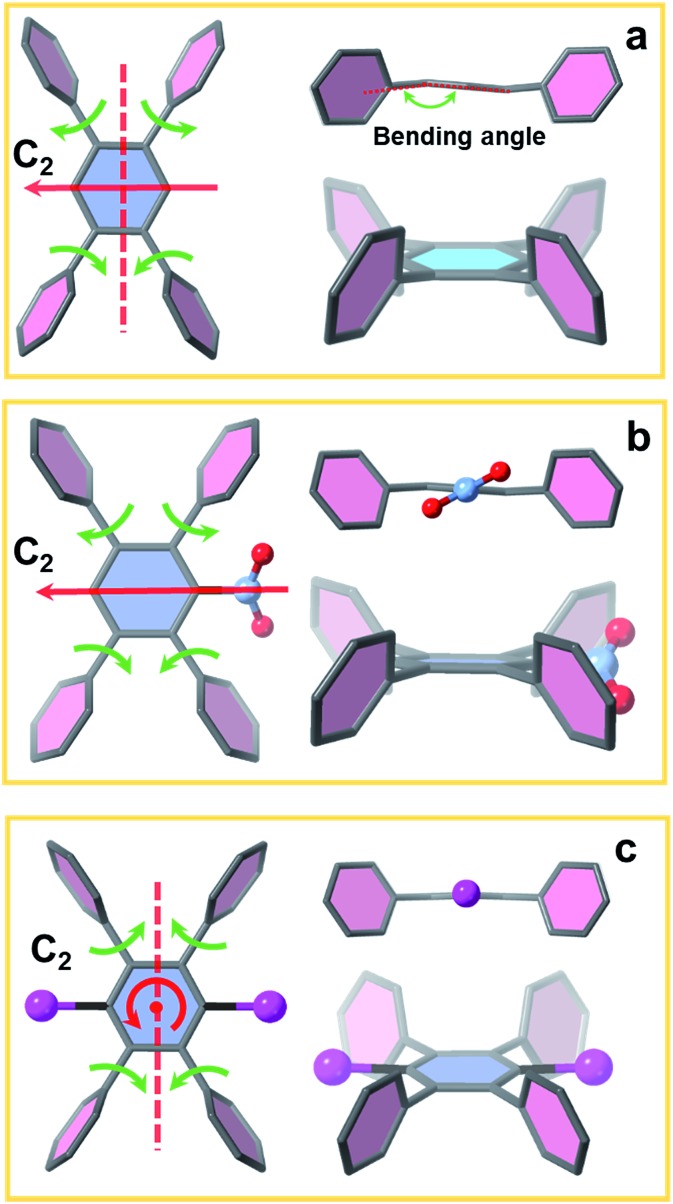
Linker conformation in (a) NU-903, (b) NU-904 and (c) NU-1008.

To investigate the influence of linker conformation on topology control, we performed a detailed analysis of linker conformation in the three crystal structures. In the **scu**-net NU-903 structure,^45^ the TCPB linker with no substituents adopts a *C*_2h_ symmetry with the *C*_2_ axis and the perpendicular *σ*_h_ depicted in Fig. 4a. In the MOF structure, the two adjacent phenyl arms in the upper side rotate away from each other, whereas the lower ones rotate toward each other (Fig. 4a). In NU-904, the introduction of the mononitro group lowers the symmetry of the linker to *C*_2_ but the conformation of the peripheral phenyl arms was found to be similar to the non-substituted TCPB linker in NU-903 (Fig. 4b). As a result, each of the twinning portions that comprise this structure has the same **scu** topology as NU-903, even though the average structure of NU-904 has 1D triangular channels instead of the diamond-shaped channels with a typical **scu** topology. Notably, the mononitro group on the central benzene rotates ∼32° to fit in the structure. In NU-1008 with dibromo-substituted TCPB, the linker adopts a *C*_2v_ symmetry due to the rotation of the upper and lower pair of phenyl arms toward each other (Fig. 4c) which directs the framework to **csq** topology over the **scu**-net MOF. In addition, to accommodate the dibromo groups in NU-1008, the dihedral angle between the arm benzene and central benzene in the **csq**-net NU-1008 is closer to 90° than the dihedral angle observed in **scu**-net NU-903 and NU-904 (Table S3, ESI†). Additionally, the angle between the arms (117°) in NU-1008 is larger than in **scu**-net NU-903 (115°) and NU-904 (108.5°).

The 8-connected Zr_6_ cluster in the three MOFs can be regarded as a cubic shaped node and the linker as a rectangular plane. In NU-903, the non-substituted TCPB linker with *C*_2h_ symmetry can link the clusters in the same orientation (Fig. 1), giving the **scu** topology. In contrast, the Zr_6_ clusters in NU-1008 undergo an orientation change to conform to the *C*_2v_ symmetry of dibromo TCPB linker, and form a different topology, **csq**. The topology change confirmed our hypothesis that the steric effect induced by linker functionalization can achieve the topology control of MOF materials.

### Catalytic performance for CO_2_ fixation under mild conditions

CO_2_ is a well-established greenhouse gas that originates from the carbon footprint of human activities.^59^ In order to mitigate the adverse effect of CO_2_ to the environment, considerable progress has been made in its capture and storage in recent years,^60^,^61^ and promising strategies for the consumption of CO_2_ have received much attention. Rational utilization of CO_2_ is of great significance for the deceleration of global warming and the development of sustainable energy. Chemical fixation of CO_2_ with epoxides through a facile cycloaddition process, catalysed by acidic sites, is an attractive route to convert this greenhouse gas into highly demanded cyclic organic carbonates.^62^

Owing to the presence of substrate accessible Lewis acidic sites, MOFs have been previously explored to catalyse the cycloaddition of CO_2_ and epoxides.^63^,^64^ However, energy-demanding reaction conditions such as elevated temperatures and pressures are generally required for the efficient conversion of CO_2_ into cyclic carbonates.^65^ Inspired by these reports, CO_2_ fixation into styrene oxide was used as a model reaction to test the catalytic performance of MOFs reported in this study under mild conditions.

**Fig. 5:**
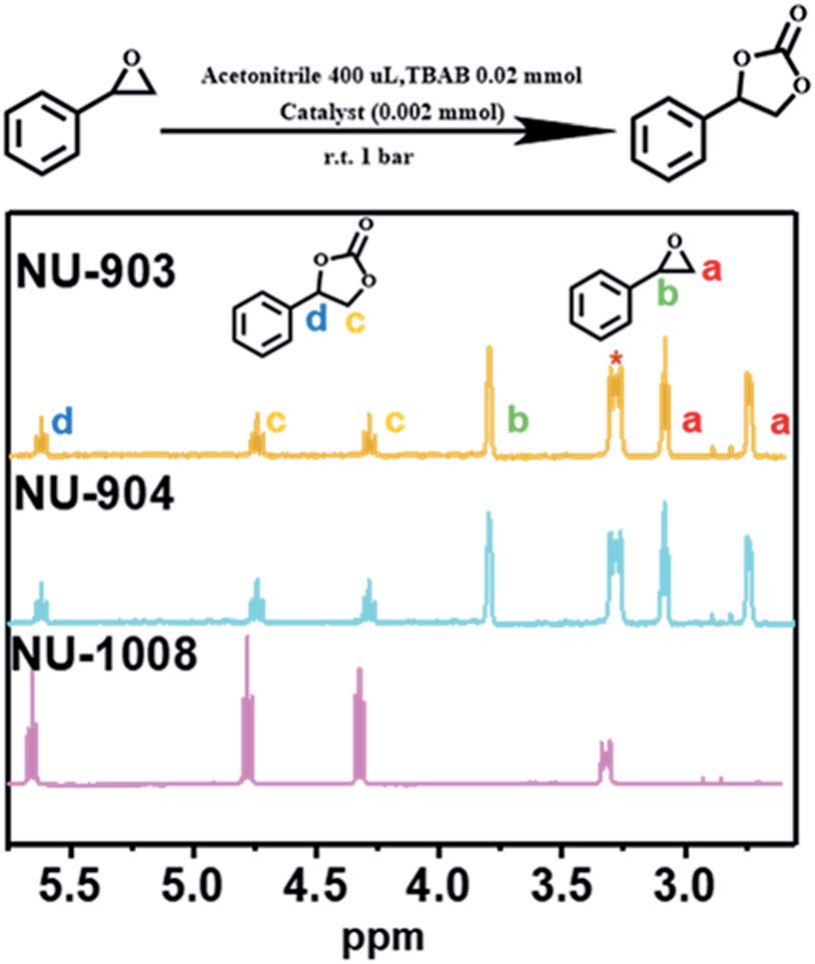
Proton NMR spectra of CO_2_ fixation into styrene oxide catalyzed by NU-903, NU-904 and NU-1008 (1 bar, room temperature, 24 h; CDCl_3_ as deuterium solvent, 500 MHz. The peak with asterisk refers to the tetrabutylammonium bromide).

NU-903, NU-904 and NU-1008 were investigated for CO_2_ fixation into styrene oxide under room temperature with 1 bar of CO_2_. The styrene oxide (0.2 mmol), tetrabutylammonium bromide (6.5 mg, 0.02 mmol) pre-dissolved in 400 μL of acetonitrile and MOF material (4.0 mol%, 0.002 mmol) were added to an autoclave batch reactor, which had previously been dried for 6 h at 80 °C. The autoclave reactor was evacuated, purged with CO_2_, and then placed under a constant pressure of CO_2_ under 5 bar for 15 min to allow the system to equilibrate. The reaction was carried out at room temperature for 24 hours after the pressure was reduced to 1 bar of gauge pressure. After the reaction, the catalyst was separated by centrifugation and a small aliquot of the supernatant reaction mixture was taken to be analyzed by ^1^H NMR to calculate the conversion (Fig. 5).

A control experiment without any MOF catalyst exhibited negligible conversion of styrene oxide. However, vastly different CO_2_ fixation activities were observed within the three MOF catalysts utilized in this report. Remarkably, full conversion of styrene oxide was observed with NU-1008 after 24 hours under mild conditions (room temperature, 1 bar of CO_2_). Previous reports using MOF catalysts for this reaction required elevated temperatures (∼120 °C), pressures (10–20 bar) or longer reaction time (up to 56 hours) to afford similar or lower substrate conversions (Table S2, ESI†).^62^ Thus, NU-1008 stands out as a more environmentally friendly solid acid catalyst for the chemical fixation of CO_2_, compared to others reported.

**Fig. 6:**
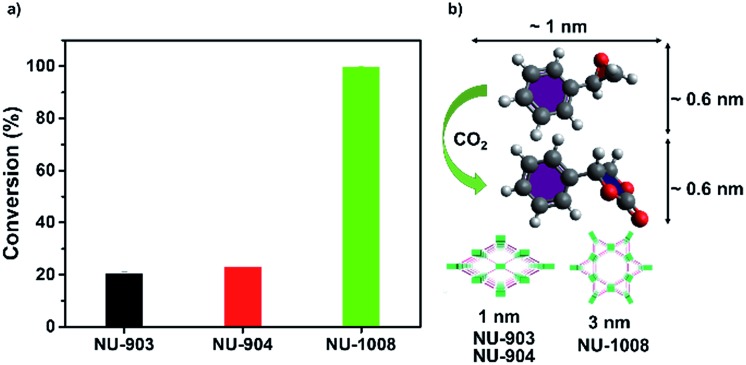
CO_2_ fixation conversion on NU-903, NU-904 and NU-1008. (a) Catalytic capacities; (b) size of substrate, product and pore size of NU-903, NU-904 and NU-1008.

In comparison, NU-903 and NU-904 showed much lower conversion (Fig. 6a), despite the fact that they had similar CO_2_ adsorption performance to NU-1008 (Fig. S15, ESI†). This significant difference is likely attributed to the aforementioned narrower pores and smaller pore volumes in NU-903 and NU-904 (around 10 Å, 0.50 cm^3^ g^–1^) compared to the mesoporous channels in NU-1008 (around 30 Å, 0.82 cm^3^ g^–1^). The microporous NU-903 and NU-904 most likely limited the diffusion of the sizeable substrate and product (Fig. 6b) and resulted in the lower conversion. The turnover numbers (TONs) in 24 hours were calculated to be 20.3, 22.8 and 99.4 for NU-903, NU-904 and NU-1008, respectively.

## Conclusions

Three different Zr-MOFs were constructed by the steric control of the conformations of substituted tetratopic carboxylate linkers. Compared to NU-903 with the **scu** topology, phase pure **csq**-net NU-1008 supports our initial hypothesis that introducing functionality into the organic linker can influence the linker conformation and direct the topology of targeted MOFs. Consequently, some mechanistic insights regarding MOF synthesis can be inferred in efforts to make MOF topology control possible with the strategic design of organic linkers. Interestingly, with nitro groups, threefold twinning is observed in **scu**-net NU-904, which yields an average structure of **shp**-net with triangular microchannels while maintaining the unsaturated metal sites for promising catalytic applications. Significantly different CO_2_ fixation catalytic activities were observed due to the diverse pore structures. Among them, the mesoporous NU-1008 is found to be a highly active MOF catalyst which displays complete CO_2_ fixation into styrene oxide in less than 24 hours under room temperature and 1 bar of CO_2_.

## Conflicts of interest

There are no conflicts to declare.

## Supplementary Material

Supplementary information

Crystal structure data
