# Supplementary material for: Topology and porosity control of metal–organic frameworks through linker functionalization
Source: Chem Sci. 2018 Nov 9;10(4):1186–92. doi: 10.1039/c8sc04220a (PMC6349059; doi:10.1039/c8sc04220a)
Supplement: Supplementary file 1 [file SC-010-C8SC04220A-s001.pdf]

Electronic Supplementary Material (ESI) for Chemical Science.
This journal is © The Royal Society of Chemistry 2018

Electronic Supplementary Information

Topology and Porosity Control of Metal–Organic Frameworks through Linker Functionalization

Jiafei Lyu,[abc] Xuan Zhang,[c] Ken-ichi Otake,[c] Xingjie Wang,[c] Peng Li,[c] Zhanyong Li,[c] Zhijie Chen,[c] Yuanyuan Zhang,[c] Megan C. Wasson,[c] Ying Yang,[c] Peng Bai,[ab] Xianghai Guo,[ab] Timur Islamoglu[c] and Omar K. Farha*[c]

[a] Department of Pharmaceutical Engineering, School of Chemical Engineering and Technology, Tianjin University, Tianjin 300350, China.
[b] Key Laboratory of Systems Bioengineering, Ministry of Education, Tianjin University, Tianjin 300350, China.
[c] Department of Chemistry and International Institute of Nanotechnology, Northwestern University, 2145 Sheridan Road, Evanston, Illinois 60208, United States.

Corresponding author e-mail: o-farha@northwestern.edu

Table of Contents

List of Figures S-1
List of Tables S-2
Section 1 Starting Materials S-3
Section 2 Ligands Synthesis S-4
Section 3 General Preparation and Activation of MOFs S-7
Section 4 Material Characterization S-9
Section 5 Single-crystal X-ray Diffraction Analyses S-17
Section 6 CO_2 Insertion Reaction S-21
Section 7 Linker Conformation in the MOFs S-22
Section 8 References S-23

List of Figures

Fig. S1 Proton NMR (500 MHz, DMSO) spectrum of 1,2,4,5-tetrakis(4-carboxyphenyl)benzene (L1). S-4
Fig. S2 Proton NMR (500 MHz, DMSO) spectrum of 1,2,4,5-tetrakis(4-carboxyphenyl)-3-nitobenzene (L2). S-5
Fig. S3 Proton NMR (500 MHz, DMSO) spectrum of 1,2,4,5-tetrakis(4-carboxyphenyl)-3,6-dibromobenzene (L3). S-6
Fig. S4 Proton NMR (500 MHz, D_2O) spectrum of NU-903. .... S-10
Fig. S5 Proton NMR (500 MHz, D_2O) spectrum of NU-904. .... S-10
Fig. S6 Proton NMR (500 MHz, D_2O) spectrum of NU-1008. .... S-11
Fig. S7 TGA curve of NU-903. .... S-11
Fig. S8 TGA curve of NU-904. .... S-12
Fig. S9 TGA curve of NU-1008. .... S-13
Fig. S10 DRIFTS spectrum of NU-903. .... S-13
Fig. S11 DRIFTS spectrum of NU-904. .... S-14
Fig. S12 DRIFTS spectrum of NU-1008. .... S-14
Fig. S13 SEM images of NU-903, NU-904 and NU-1008. .... S-15
Fig. S14 PXRD patterns of NU-903, NU-904 and NU-1008 after treatment of 0.5 M HCl aqueous solution S-15
Fig. S15 CO_2 adsorption of NU-903, NU-904 and NU-1008 at 298 K. S-16
Fig. S16 Directly observed Zr_{18} cluster and their coordinating OH/OH_2 groups (left) and overlapping Zr_6 clusters, each exhibiting partial crystallographic occupancy of 1/3(right). S-18
Fig. S17 The topological net of the ordered structure of NU-904 (a and b); and the topological net of threefold twinned structure of NU-904 (c and d). S-19
Fig. S18 The fragments of the three different frameworks are depicted with octahedron Zr_6 cluster and benzene planes in the organic linkers. (a) NU-903. (b) NU-904. (c) NU-1008. .... S-22

List of Tables

Table S1. Summary of the crystal data and structure refinement details for NU-1008 and NU-904 at 200 K..S-20
Table S2 Comparison of CO_2 fixation performance with other MOF materials......S-21
Table S3 The bending angle between center benzene plane and peripheral arms benzene (ψ), the dihedral angles of the arm benzene to the center benzene (ϕ) and those of the carboxylate to the arm benzene (δ), angles between arm benzenes (α/α') and the angle of nitro group to center benzene (γ) measured from single crystal structures of three MOFs. S-22

Section 1 Starting Materials

All chemicals and solvents were purchased from commercial suppliers and used without further purification. Zirconium(IV) oxynitrate hydrate (99%), formic acid (≥96%), 1,2,4,5-tetrakis(4-carboxyphenyl)benzene (TCPB) (≥98%), zirconium chloride (≥99.5%), p-tolylmagnesium bromide solution (1.0 M in DMF), hexabromobenzene (98%), 1,2,4,5-tetrabromobenzene (97%), bromine (reagent grade), carbon tetrachloride (99.9%), sodium nitrate (≥99%), benzene (99.8%), potassium carbonate (99%), *N,N*-diethylformamide (DEF) and 4-ethoxycarbonylphenylboronic acid (98%) were purchased from Sigma-Aldrich. *N,N*-dimethylformamide (DMF) (99.9%), acetone (99.8%), hydrochloric acid (36.5-38%), nitric acid (67-70%), chloroform (99.8%), sulfuric acid (95-98%), sodium hydroxide (≥97%), anhydrous ethanol (95%), palladium tetraphenylphosphine (99%), dichloromethane (99.9%), methanol (99.9%) and hexane (98.5%) were purchased from Fisher Chemical. Deionized water was used as the water source throughout the experiments.

Section 2 Ligands Synthesis

Synthesis of 1,2,4,5-tetrakis(4-carboxyphenyl)benzene (L1)[1]

(a) 100 mL of p-tolylmagnesium bromide (1 M in THF, 100 mmol) was added under nitrogen to a flask containing 5 g of hexabromobenzene (9.07 mmol). The mixture was stirred at room temperature for 15 h. The reaction was poured on ice then 50 mL of 6 M HCl was added. The mixture was extracted with THF (3 x 200 mL). The organics were combined and the solvent was removed via rotary evaporation. The solid was then washed with hexanes and cold acetone. Isolated yield: 2.75g, 70% based on hexabromobenzene.

(b) An amount of 2 g from (a) was placed in a 100 mL teflon lined vessel. An amount of 24 mL of water and 6 mL of HNO_3 were then added. The vessel was sealed and heated at 180 °C for 24 h. The resulting solid was collected by filtration and washed with THF/$CHCl_3$ (7:3). Isolated yield: 1.85g, 75%.

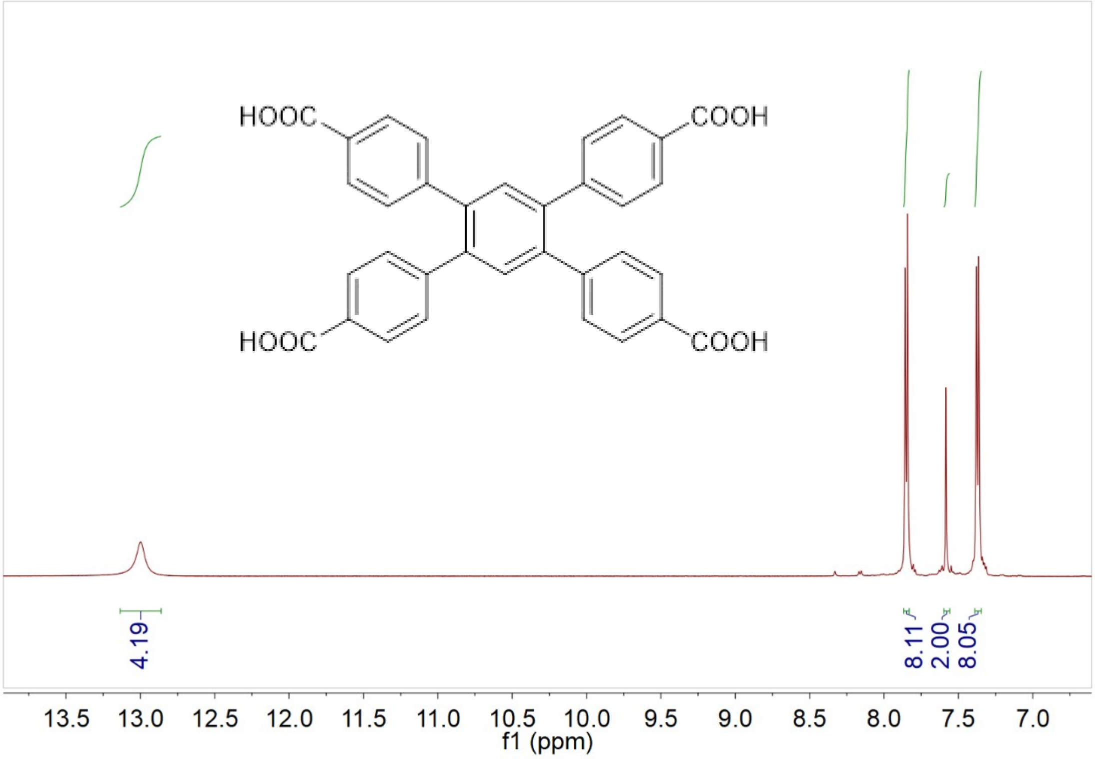

Fig. S1 Proton NMR (500 MHz, DMSO) spectrum of 1,2,4,5-tetrakis(4-carboxyphenyl)benzene (L1).

Synthesis of 1,2,4,5-tetrakis(4-carboxyphenyl- 3-nitrobenzene (L2)[2]

(a) A mixture of 1,2,4,5-tetrabromobenzene (3.7 g), $NaNO_3$ (1.8 g), and conc. H_2SO_4 (10 mL) was heated on a water bath with stirring for 7 h. The reaction mixture was cooled, poured onto ice, and neutralized with NaOH. The precipitate that formed was filtered off, washed with water, dried, and recrystallized from benzene.

(b) To a 500 mL round bottom flask, (a) (4.39 g), 4-ethoxycarbonylphenylboronic acid (23.28 g), potassium carbonate (16.6 g), 210 mL toluene, 21 mL ethanol and 21 mL water were added. The mixture was purged with nitrogen for 30 min then

palladium tetraphenylphosphine (800 mg) was added into the mixture. The mixture was further purged with nitrogen for another 15 min, then heated at 110 °C for 65 h. After the reaction was done, the solid impurity was filtered off and the filtrate was evacuated under vacuum. The rough product was extracted with dichloromethane and water. The organic layer was evacuated and the obtained solid was washed with methanol to get pure product. Isolated yield: 4.59g, 64%.

(c) 1 g (b) was added into a mixture of 30 mL THF, 30 mL methanol and 23 mL 10 M NaOH solution. The mixture was refluxed overnight to be completely hydrolyzed. Concentrated hydrogen chloride was slowly added into the reaction (dropwise) with stirring through an addition funnel until the solution had reached a pH of 1. The formed white precipitate was collected, washed with water and dried. Isolated yield: 600 mg, 71%.

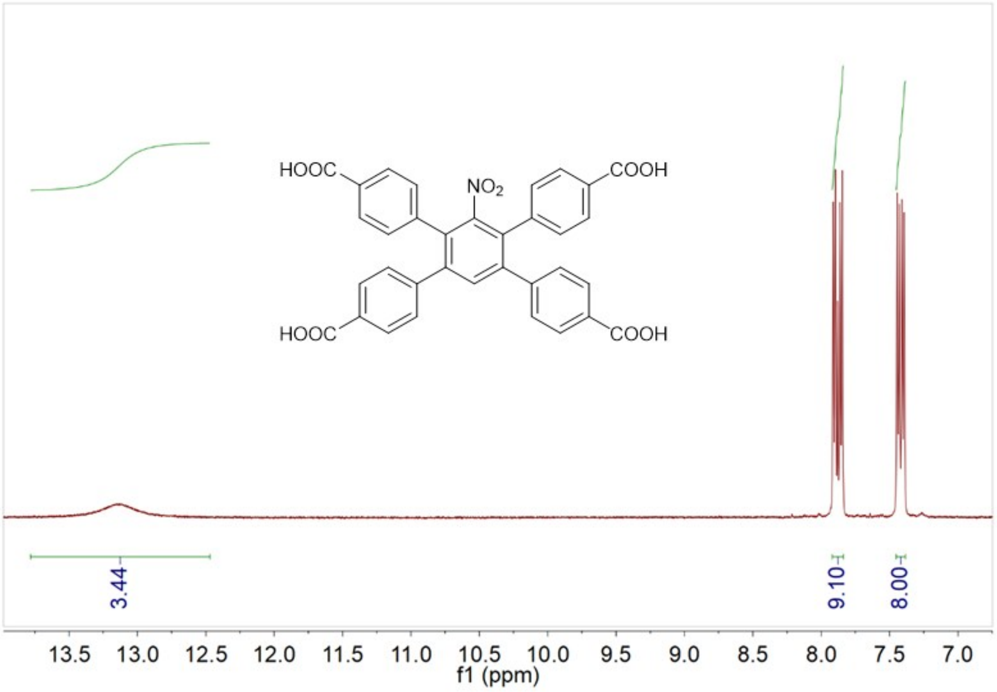

Fig. S2 Proton NMR (500 MHz, DMSO) spectrum of 1,2,4,5-tetrakis(4-carboxyphenyl)-3-nitobenzene (L2).

Synthesis of 1,2,4,5-tetrakis(4-carboxyphenyl)- 3,6-dibromobenzene (L3)[3]

(a) 100 mL of p-tolylmagnesium bromide (1 M in THF, 100 mmol) was added under nitrogen to a flask containing 5g of hexabromobenzene (9.07 mmol). The mixture was stirred at room temperature for 15 h (gray suspension). The reaction was placed on an ice-bath. A mixture of 7 mL of bromine and 60 mL of CCl_4 were placed in an addition funnel and then added drop wise to the reaction mixture. The reaction was stirred at room temperature for 1.5 h and then poured on ice, which was followed by the addition of 50 mL of 6 M HCl. The solid was filtered and washed with methanol. Isolated yield: 2.25 g, 42 %.

(b) An amount of 2 g from (a) was placed in a 100 mL teflon lined vessel. An amount of 24 mL of water and 6 mL of HNO_3 were then added. The vessel was sealed and heated at 180 °C for 24 h. The resulting solid was collected by filtration and washed

with THF/$CHCl_3$ (7:3). Isolated yield: 1.85g, 75%.

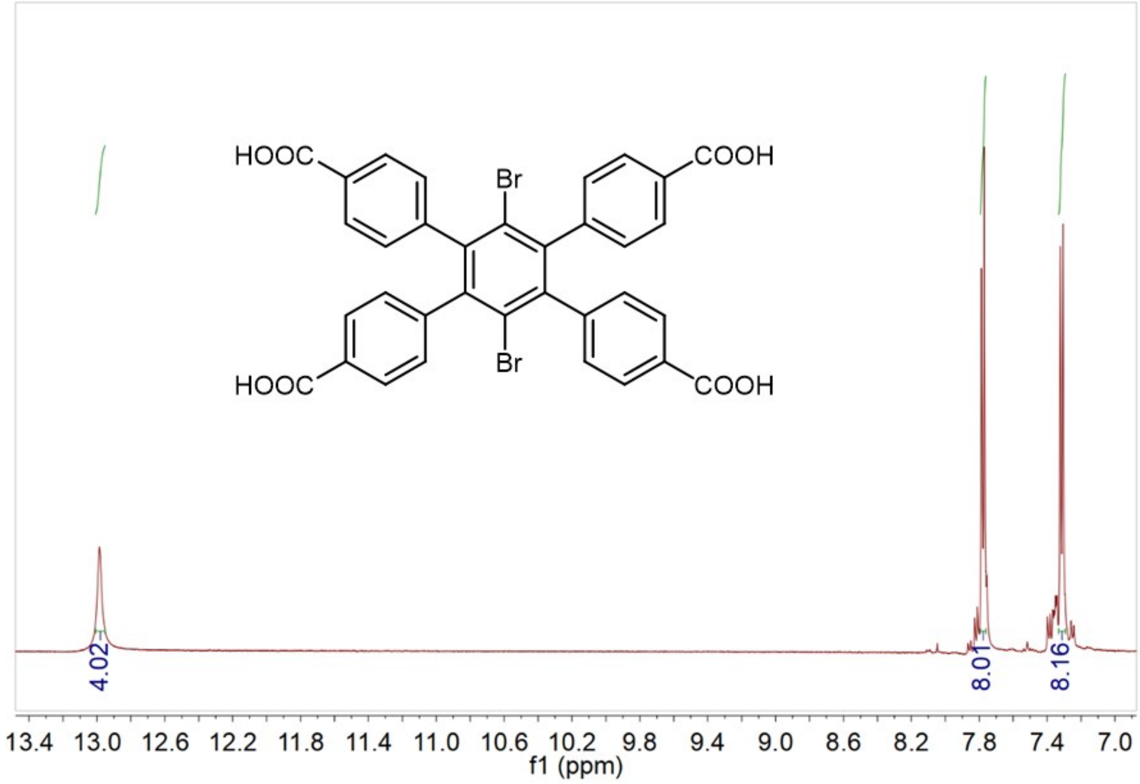

Fig. S3 Proton NMR (500 MHz, DMSO) spectrum of 1,2,4,5-tetrakis(4-carboxyphenyl)-3,6-dibromobenzene (L3).

Section 3 General Preparation and Activation of MOFs

NU-903. Zirconium oxynitrate hydrate (0.267 mmol, 66.4 mg) and 1 mL formic acid were added to *N,N*-dimethylformamide (1.5 mL) to obtain the Zr_6 cluster solution by heating the mixture at 80 °C for 1 h. After cooling down the mixture, a solution prepared by dissolving L1 (0.0533 mmol, 29.8 mg) in 1.5 mL DMF was added into the prepared node solution. The mixture was heated under 100 °C overnight (15-16 h). The generated white powder was centrifuged out of the solution and washed with *N,N*-dimethylformamide (5 mL×3) and acetone (5 mL×3). Then the material was soaked in acetone overnight and washed with acetone for another three times. Discarding the supernatant after centrifuging, the material was dried under vacuum at 80 °C for 1 h.

NU-904 Powder. Zirconium oxynitrate hydrate (0.021 mmol, 5 mg) and 25 μL formic acid were added to *N,N*-dimethylformamide (75 μL) to obtain the Zr_6 cluster solution by heating the mixture at 80 °C for 1 h. After cooling down the mixture, a solution prepared by dissolving L2 (1.34 μmol, 2 mg) in 75 μL *N,N*-dimethylformamide was added into the prepared node solution. Additional 175 μL formic acid was added. The mixture was heated under 100 °C overnight (15-16 h). The generated powder was centrifuged out of the solution and washed with *N,N*-dimethylformamide (5 mL×3) and acetone (5 mL×3). Then the material was soaked in acetone overnight and washed with acetone for another three times. Discarding the supernatant after centrifuging, the material was dried under vacuum at 80 °C for 1 h.

NU-904 Single Crystal. Zirconium chloride (0.008 mmol, 1.87 mg) and 30 μL formic acid were added to *N,N*-diethylformamide (0.5 mL) to obtain the Zr_6 cluster solution by heating the mixture at 80 °C for 1 h. After cooling down the mixture, a solution prepared by dissolving L2 (1.34 μmol, 0.81 mg) in 0.5 mL *N,N*-diethylformamide was added into the prepared node solution. Additional 0.55 mL formic acid was added. After heated under 120°C for 3 days, football-shaped single crystals formed.

NU-1008 Powder. Zirconium oxynitrate hydrate (0.267 mmol, 66.4 mg) and 1 mL formic acid were added to *N,N*-dimethylformamide (1.5 mL) to obtain the Zr_6 cluster solution by heating the mixture at 80 °C for 1 h. After cooling down the mixture, a solution prepared by dissolving L3 (0.0533 mmol, 38.2 mg) in 1.5 mL *N,N*-dimethylformamide was added into the prepared node solution. The mixture was heated under 100°C overnight (15-16 h). The generated white powder was centrifuged out of the solution and washed with *N,N*-dimethylformamide (5 mL×3) and acetone (5 mL×3). Then the material was soaked in acetone overnight and washed with acetone for another three times. Discarding the supernatant after centrifuging, the material was dried under vacuum at 80 °C for 1 h.

NU-1008 Single Crystal. Zirconium chloride (0.008 mmol, 1.87 mg) and 30 μL formic acid were added to *N,N*-diethylformamide (0.5 mL) to obtain the Zr_6 cluster solution by heating the mixture at 80 °C for 1 h. After cooling down the mixture, a solution prepared by dissolving L3 (0.0013 mmol, 0.965 mg) in 0.5 mL *N,N*-diethylformamide was added into the prepared node solution. Additional 0.45 mL formic acid was added. After heated under 120°C for 3 days, rod-shaped single crystals formed.

Activation of MOFs. After washing with *N,N*-dimethylformamide and acetone, three freshly synthesized MOFs were activated by heating at 120 ˚C for overnight under high vacuum on a Micromeritics Smart Vacprep.

Section 4 Material Characterization

X-ray Diffraction Analyses. Powder x-ray diffraction (PXRD) patterns were recorded on a Rigaku X-ray Diffractometer Model SmartLab equipped with an 18 kW Cu rotating anode, an MLO monochromator, and a high-count-rate scintillation detector. Measurements were made over the range 1 °$<2\theta<$ 30 ° in 0.05 ° step width with a 5 °/min scanning speed.

N_2 Sorption Measurements. N_2 adsorption and desorption isotherms on activated materials were measured on a Micromeritics ASAP 2020 (Micromeritics, Norcross, GA) instrument at 77 K. Around 30 mg of sample was used in each measurement and the specific surface areas were determined using the Brunauer−Emmett−Teller model from the N_2 sorption data in the region P/P_0 = 0.005–0.05. Pore size distributions were obtained using DFT calculations using a carbon slit-pore model with a N_2 kernel.

Proton NMR. MOF samples (1 mg) were digested with NaOD solution (0.1 M in deuterium oxide, 150 μL). After sonication for 20 min, another 500 μL of deuterium oxide was added into the mixture to dilute the solution. Proton NMR spectra were collected on a Bruker Avance III 500 MHz system equipped with DCH CryoProbeand automated with a BACS-60 autosampler.

Diffuse Reflectance for Infrared Fourier Transform Spectroscopy (DRIFTS). DRIFTS spectra were recorded on a Nicolet 6700 FTIR spectrometer equipped with an MCT detector. The detector was cooled with liquid N_2 and the spectra were collected under Ar atmosphere. KBr was utilized as a background spectrum.

Thermogravimetric Analyses (TGA). TGA was performed on a TGA/DCS 1 system (Mettler-Toledo AG, Schwerzenbach, Switzerland), which runs on a PC with STARe software. Samples were heated from 25 to 900 °C at a rate of 10 °C/min under air with flow rate 20 mL/min.

CO_2 Sorption Measurement. CO_2 adsorption and desorption isotherms on activated materials were measured on a Micromeritics ASAP 2020 (Micromeritics, Norcross, GA) instrument at 298 K in water bath.

Stability under Acidic Condition. The stability of materials was tested by soaking the materials in 0.5 M HCl aqueous solution for 10 h. The crystallinity of materials was characterized by X-ray diffraction analyses after decanting the soaking solution.

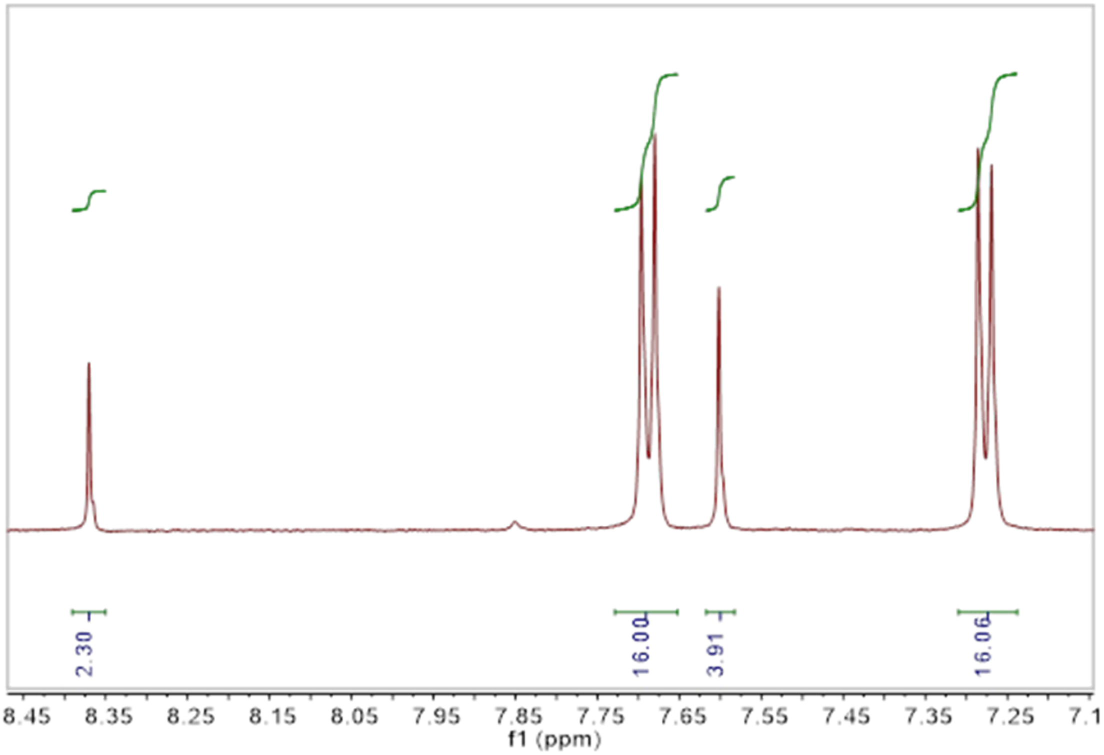

Fig. S4 Proton NMR (500 MHz, D_2O) spectrum of NU-903.

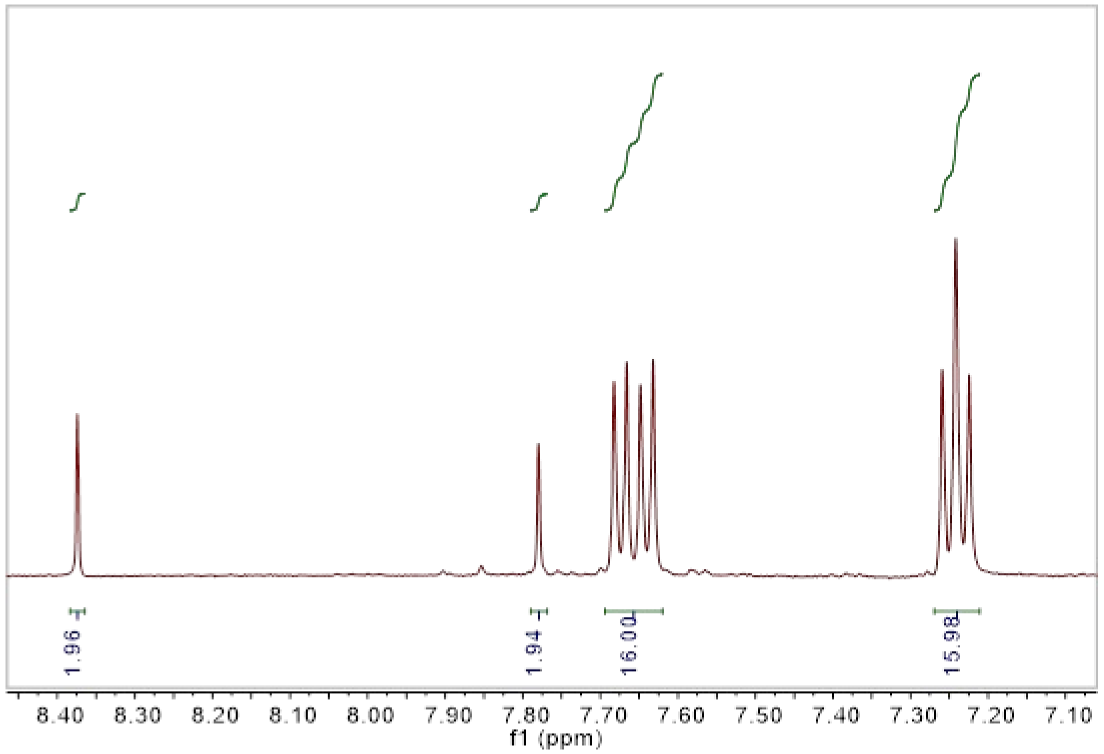

Fig. S5 Proton NMR (500 MHz, D_2O) spectrum of NU-904.

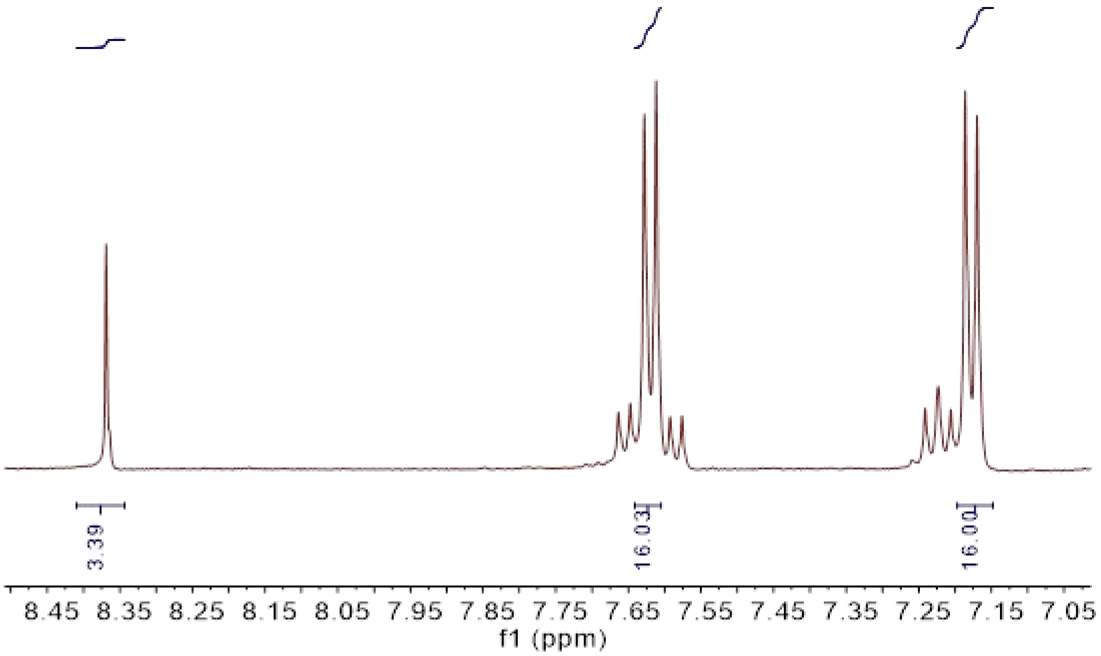

Fig. S6 Proton NMR (500 MHz, D_2O) spectrum of NU-1008.

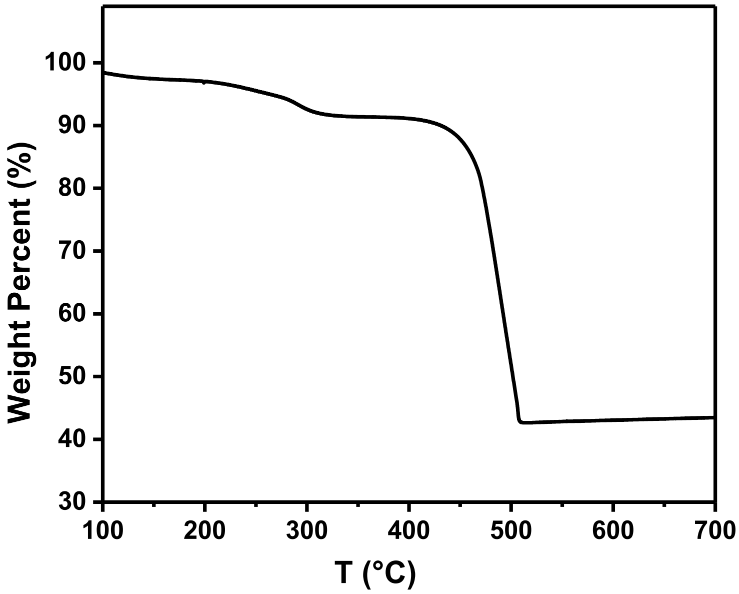

Fig. S7 TGA curve of NU-903.

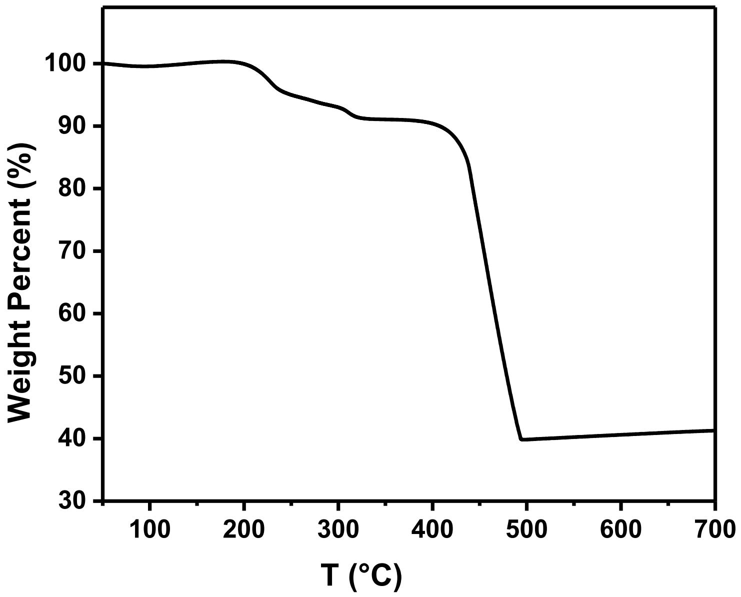

Fig. S8 TGA curve of NU-904.

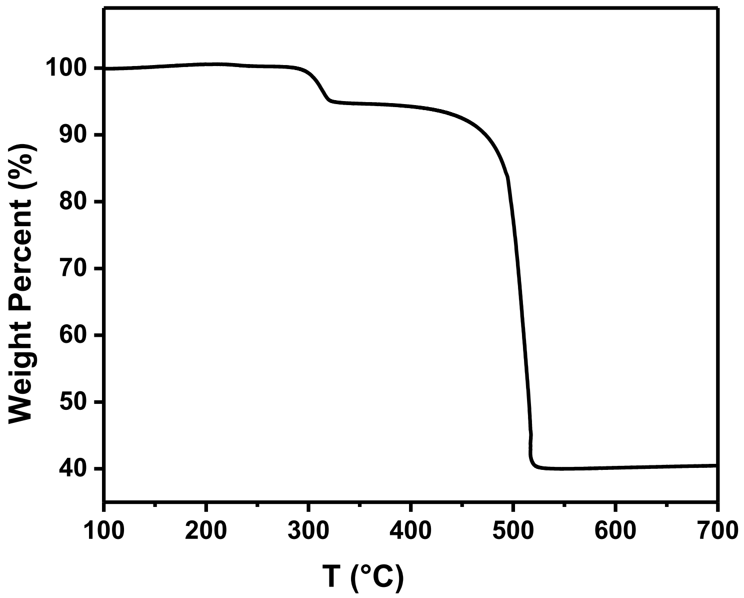

Fig. S9 TGA curve of NU-1008.

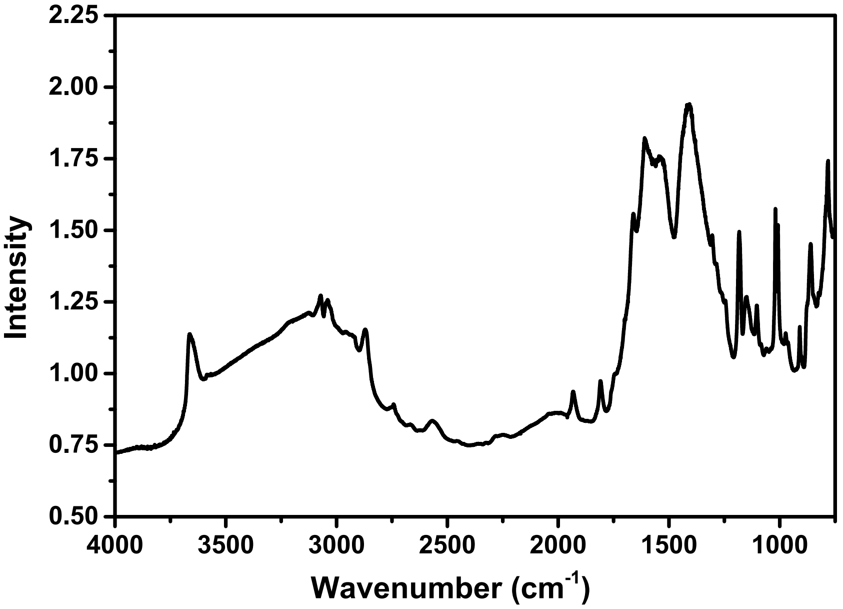

Fig. S10 DRIFTS spectrum of NU-903.

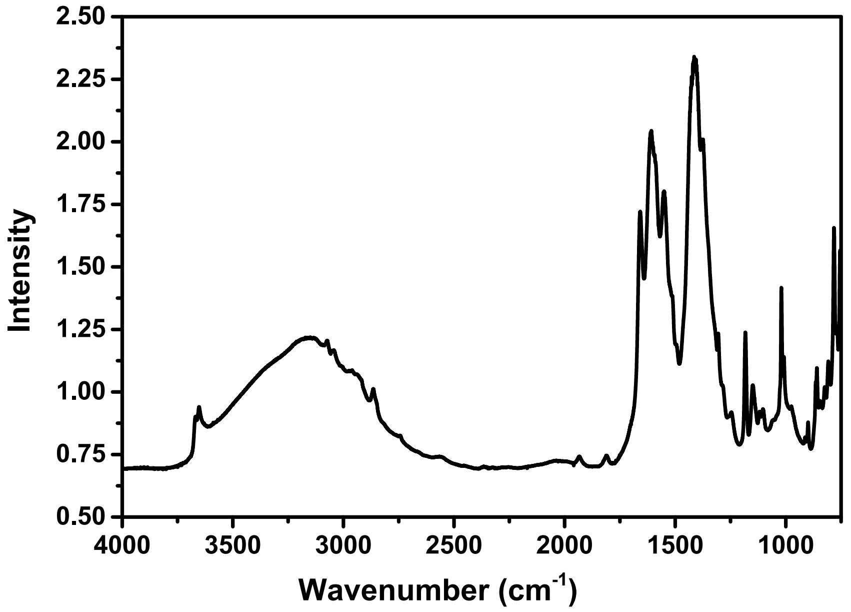

Fig. S11 DRIFTS spectrum of NU-904.

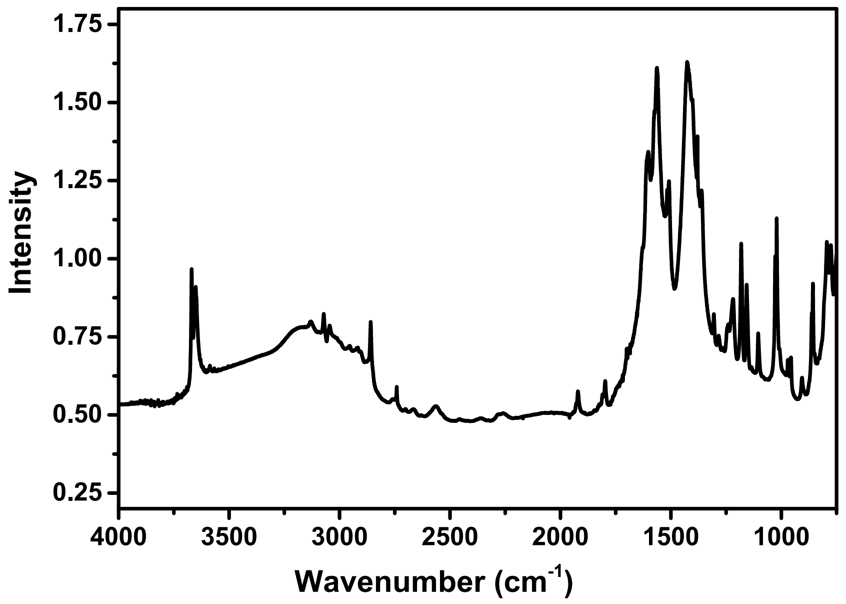

Fig. S12 DRIFTS spectrum of NU-1008.

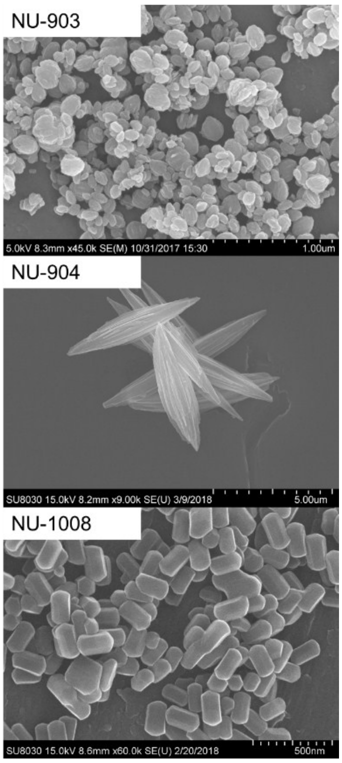

Fig. S13 SEM images of NU-903, NU-904 and NU-1008.

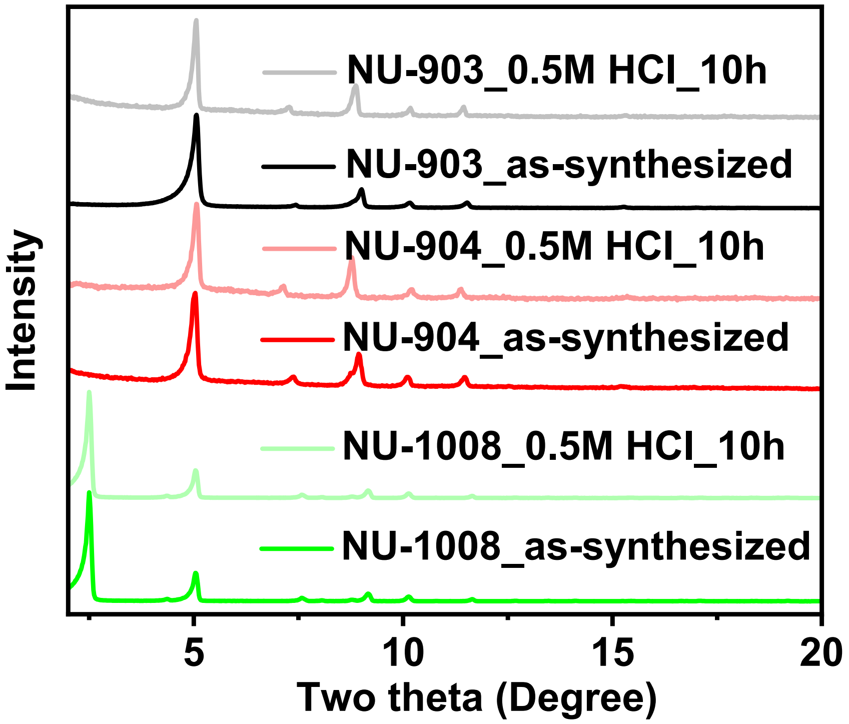

Fig. S14 PXRD patterns of NU-903, NU-904 and NU-1008 after treatment of 0.5 M HCl aqueous solution.

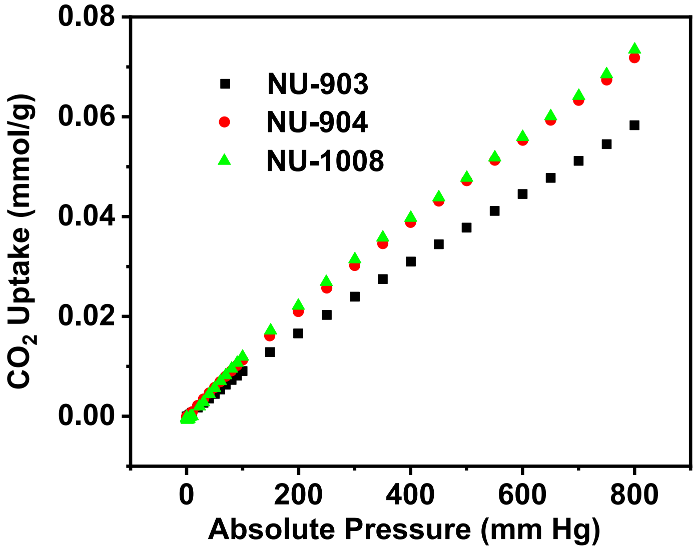

Fig. S15 CO_2 adsorption of NU-903, NU-904 and NU-1008 at 298 K.

Section 5 Single-crystal X-ray Diffraction Analyses

Single crystals of NU-1008 and NU-904 were mounted on MicroMesh (MiTeGen) with paraton oil. The data was collected on a 'Bruker APEX-II CCD' diffractometer with a Mo or Cu Kα microfocus X-ray source. The crystals were kept at the desired temperatures during data collection. Using Olex2 [2] (for NU-1008) or Yadokari-XG (for NU-904) softwares[4], the structure was solved with the ShelXT [3] structure solution program using Intrinsic Phasing and refined with the ShelXL [4] refinement package using Least Squares minimization. The disordered non-coordinated solvents were removed using the PLATON SQUEEZE program. The refinement results are summarized in Table S1. Crystallographic data for the NU-1008 and NU-904 crystal structures in CIF format has been deposited in the Cambridge Crystallographic Data Centre (CCDC) under deposition numbers CCDC-1855836 (NU-1008) and 1854453 (NU-904). The data can be obtained free of charge via www.ccdc.cam.ac.uk/data_request/cif (or from the Cambridge Crystallographic Data Centre, 12 Union Road, Cambridge CB2 1EZ, U.K.)

Refinement Detail of NU-904

We firstly attempted to solve the structure with the hexagonal cell, which indicated Zr_{18} clustered MOF structure. However, the least-squares refinement of the crystal structure did not converge. As similar to the previous case of PCN-223[5], the data should be interpreted as the twinning with reticular merohedry[6], and the observed Zr_{18} cluster can be refined as three overlaps of Zr_6 cluster with different orientation (Fig. S14). Because the coordinating OH/OH_2 groups pointing along *c*-axis were clearly observed, we interpreted the structure as the 8-connected Zr_6 cluster MOF structure, instead of 12-connected cluster MOF structure. By lowering the symmetry to *monoclinic P* and using PART command (PART 1, 2, 3), the twinning structures was successfully divided into 3 components with the same occupancies (1/3). Some structural and thermal parameter restraints (DFIX, DANG, SADI, FLAT, RIGU, SIMU, ISOR) were used in the refinements. And one thermal parameter constraint (EADP) on bridged oxygens (O2X, O2Y, O2Z) on Zr_6 node was also used in the refinement.

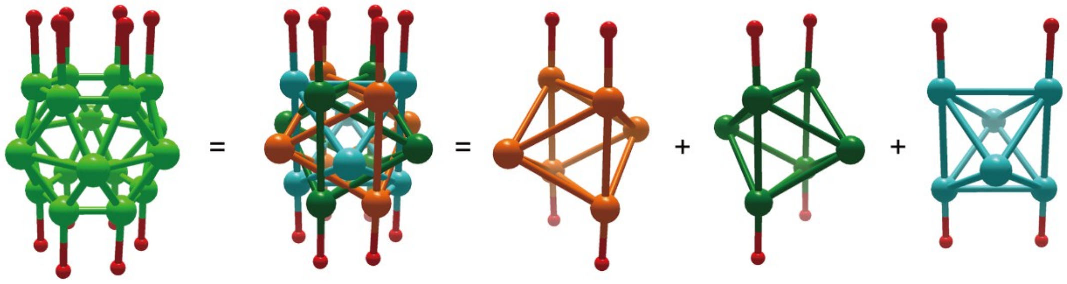

Fig. S16 Directly observed Zr_{18} cluster and their coordinating OH/OH_2 groups (left) and overlapping Zr_6 clusters, each exhibiting partial crystallographic occupancy of 1/3(right).

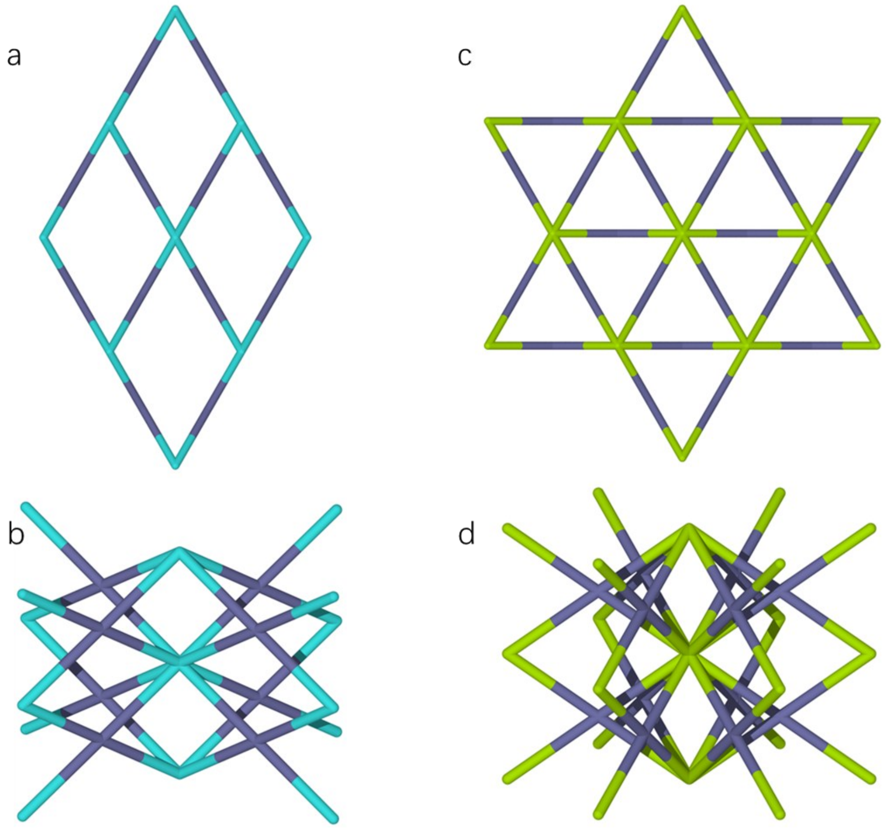

Fig. S17 The topological net of the ordered structure of NU-904 (a and b); and the topological net of threefold twinned structure of NU-904 (c and d).

Table S1. Summary of the crystal data and structure refinement details for NU-1008 and NU-904 at 200 K.

	NU-1008	NU-904
Empirical formula	$C_{69}H_{32}Br_4O_{32}Zr_6$	$C_{69.5}H_{32}N_2O_{36}Zr_6$
Formula weight / (g/mol)	2239.90	2018.29
Temperature/K	200(2)	200(2)
Crystal system	hexagonal	monoclinic
Space group	P6/mmm	P2/m
a/Å	39.5745(12)	19.6381(9)
b/Å	39.5745(12)	12.6310(6)
c/Å	12.1936(4)	19.6354(9)
α/°	90	90
β/°	90	119.994(2)
γ/°	120	90
Volume/Å^3	16538.4(11)	4218.3(3)
Z	3	1
ρ_{calc}g/cm^3	0.675	0.795
μ/mm^{-1}	1.028	3.304
F(000)	3246.0	991.0
Crystal size/mm^3	0.07 × 0.03 × 0.02	0.07 × 0.09 × 0.18
Radiation	MoKα (λ = 0.71073)	CuKα (λ = 1.54178)
2Θ range for data collection/°	1.188 to 54.34	5.196 to 122.77
Index ranges	-50 ≤ h ≤ 50, -50 ≤ k ≤ 50, -15 ≤ l ≤ 10	-22 ≤ h ≤ 22, -13 ≤ k ≤ 14, -22 ≤ l ≤ 22
Reflections collected	296742	34163
Independent reflections	6868 [R_{int} = 0.0911, R_{sigma} = 0.0214]	6705 [R_{int} = 0.0590, R_{sigma} = 0.0502]
Data/restraints/parameters	6868/0/141	6705/3011/721
Goodness-of-fit on F^2	1.076	1.166
Final R indexes [I>=2σ (I)]	R_1 = 0.0445, wR_2 = 0.1435	R_1 = 0.1254, wR_2 = 0.3343
Final R indexes [all data]	R_1 = 0.0510, wR_2 = 0.1492	R_1 = 0.1320, wR_2 = 0.3384
Largest diff. peak/hole / e Å^{-3}	1.71/-1.43	0.55/ -1.46

Section 6 CO_2 Insertion Reaction

Batch reactions were carried out by using NU-903, NU-1008 and NU-904 as catalysts for CO_2 insertion into styrene oxide. The styrene oxide (0.2 mmol), tetrabutylammonium bromide (6.5 mg, 0.02 mmol) pre-dissolved in 400 μL of acetonitrile and MOF material (4.0 mol %) were added to an autoclave reactor, which had previously been dried for 6 h at 80 °C. The autoclave reactor was evacuated, purged with CO_2, and then placed under a constant pressure of CO_2 under 5 atm for 15 min to allow the system to equilibrate. Then the pressure was reduced to 1 atm of gauge pressure and the reaction was carried out at room temperature for 24 h. At the end of the reaction, the reactor was placed in an ice bath for 10 min and then opened. After catalyst separation by centrifugation, a small aliquot of the supernatant reaction mixture was taken to be analyzed by ^{1}H NMR to calculate the conversion of the reaction.

Table S2 Comparison of CO_2 fixation performance with other MOF materials.

Entry	MOFs	Temperature (°C)	Pressure (bar)	Time (h)	Yield (%)	References
1	Zr-NU-903	R.T.	1	24	20	This study
2	Zr-NU-904	R.T.	1	24	23	This study
3	Zr-NU-1008	R.T.	1	24	99.5	This study
4	Blank	R.T.	1	24	0	This study
5	gea-MOF-1	120	20	6	85	[7]
6	Co-MOF-74	100	20	4	96	[8, 9]
7	Mg-MOF-74	100	20	4	94	[10]
8	Cr-MIL-101	25	8	48	95	[9]
9	Fe-MIL-101	25	8	48	93	[11]
10	HKUST-1	100	20	4	48	[10]
11	Ni(salphen)-MOF	80	20	4		[12]
12	MIL-68(In)	150	8	8	39	[13]
13	MIL-68(In)-NH_2	150	8	8	71	[13]
14	UiO-66	100	20	1	48	[10]
15	UiO-66-NH_2	100	20	1	70	[10]
16	F-IRMOF-3	140	20	5	84	[14]
17	IRMOF-3	100	20	4	33	[10]
18	Hf-NU-1000	R.T.	1	56	100	[15]
19	Zr-NU-1000	R.T.	1	56		[15]
20	ZIF-8	100	7	5	54	[16]
21	ZIF-68	120	10	12	93	[17]

Section 7 Linker Conformation in the MOFs

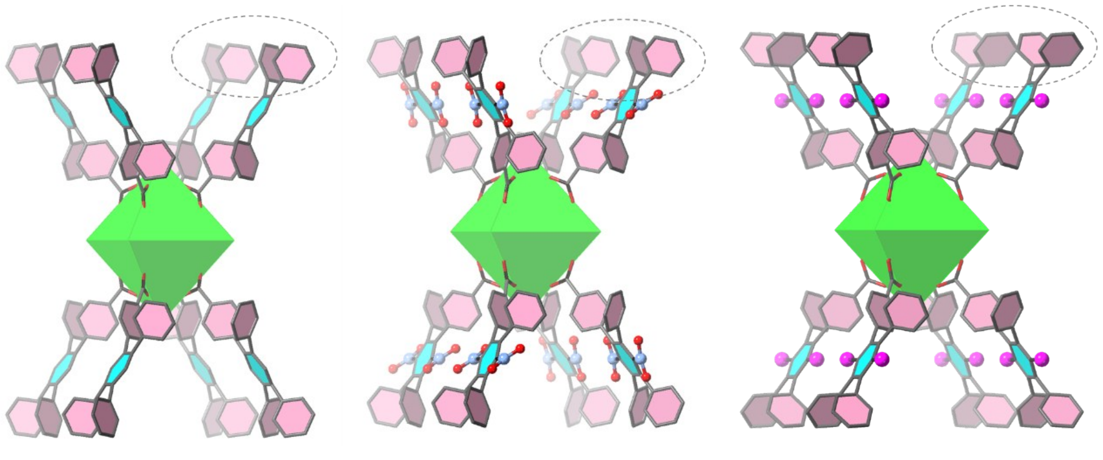

a b c

Fig. S18 The fragments of the three different frameworks are depicted with octahedron Zr_6 cluster and benzene planes in the organic linkers. (a) NU-903. (b) NU-904. (c) NU-1008.

Table S3 The bending angle between center benzene plane and peripheral arms benzene (*ψ*), the dihedral angles of the arm benzene to the center benzene (*ϕ*) and those of the carboxylate to the arm benzene (*δ*), angles between arm benzenes (*α/α'*) and the angle of nitro group to center benzene (*γ*) measured from single crystal structures of three MOFs.

MOFs	*ψ*	*ϕ*	*δ*	*α/α'*		*γ*
NU-903	144.27	110.19/110.19/69.82/69.82	26.02/26.02/-26.02/-26.02	62.7	114.62	-
NU-904	147.31	117.67/117.67/62.33/62.33	9.8/9.8/-9.8/-9.8	69.88	108.5	32.15
NU-1008	178.36	102.36/77.65/102.36/77.65	23.13/-3.13/23.13/-3.13	62.64	117.22	-

Section 8 References

1. O. K. Farha, K. L. Mulfort and J. T. Hupp, *Inorg. Chem.*, 2008, **47**, 10223-10225.
2. V. N. Shishkin, I. V. Tarasova and K. P. Butin, *Russ. Chem. Bull.*, 2005, **54**, 2379-2383.
3. O. K. Farha, C. D. Malliakas, M. G. Kanatzidis and J. T. Hupp, *J. Am. Chem. Soc.*, 2010, **132**, 950-952.
4. C. A. Kabuto, S.; Nemoto, T.; Kwon, E., *J. Cryst. Soc. Jpn.*, 2009, **51**, 218-224.
5. D. Feng, Z.-Y. Gu, Y.-P. Chen, J. Park, Z. Wei, Y. Sun, M. Bosch, S. Yuan and H.-C. Zhou, *J. Am. Chem. Soc.*, 2014, **136**, 17714-17717.
6. S. Parsons, *Acta Crystallogr., Sect. D: Biol. Crystallogr.*, 2003, **59**, 1995-2003.
7. V. Guillerm, Ł. J. Weseliński, Y. Belmabkhout, A. J. Cairns, V. D'Elia, Ł. Wojtas, K. Adil and M. Eddaoudi, *Nature Chem.*, 2014, **6**, 673.
8. H.-Y. Cho, D.-A. Yang, J. Kim, S.-Y. Jeong and W.-S. Ahn, *Catal. Today*, 2012, **185**, 35-40.
9. O. V. Zalomaeva, A. M. Chibiryaev, K. A. Kovalenko, O. A. Kholdeeva, B. S. Balzhinimaev and V. P. Fedin, *J. Catal.*, 2013, **298**, 179-185.
10. J. Kim, S.-N. Kim, H.-G. Jang, G. Seo and W.-S. Ahn, *Appl. Catal., A*, 2013, **453**, 175-180.
11. O. V. Zalomaeva, N. V. Maksimchuk, A. M. Chibiryaev, K. A. Kovalenko, V. P. Fedin and B. S. Balzhinimaev, *J. Energy Chem.*, 2013, **22**, 130-135.
12. Y. Ren, Y. Shi, J. Chen, S. Yang, C. Qi and H. Jiang, *RSC Adv.*, 2013, **3**, 2167-2170.
13. T. Lescouet, C. Chizallet and D. Farrusseng, *ChemCatChem*, 2012, **4**, 1725-1728.
14. X. Zhou, Y. Zhang, X. Yang, L. Zhao and G. Wang, *J. Mol. Catal. A: Chem.*, 2012, **361-362**, 12-16.
15. M. H. Beyzavi, R. C. Klet, S. Tussupbayev, J. Borycz, N. A. Vermeulen, C. J. Cramer, J. F. Stoddart, J. T. Hupp and O. K. Farha, *J. Am. Chem. Soc.*, 2014, **136**, 15861-15864.
16. C. M. Miralda, E. E. Macias, M. Zhu, P. Ratnasamy and M. A. Carreon, *ACS Catal.*, 2012, **2**, 180-183.
17. L. Yang, L. Yu, G. Diao, M. Sun, G. Cheng and S. Chen, *J. Mol. Catal. A: Chem.*, 2014, **392**, 278-283.
